# Intestinal Organoids Generated from Human Pluripotent Stem Cells

**DOI:** 10.31662/jmaj.2019-0027

**Published:** 2019-12-27

**Authors:** Satoru Tsuruta, Hajime Uchida, Hidenori Akutsu

**Affiliations:** 1 Department of Gastroenterological Surgery, Hirosaki University Graduate School of Medicine, Hirosaki, Japan; 2 Centre for Regenerative Medicine, National Research Institute for Child Health and Development, Tokyo, Japan; 3 Transplantation Centre, National Centre for Child Health and Development, Tokyo, Japan

**Keywords:** intestinal organoids, embryonic stem cells, induced pluripotent stem cells, gastrointestinal disease, drug discovery

## Abstract

The gastrointestinal system is one of the most complex organ systems in the human body, and consists of numerous cell types originating from three germ layers. To understand intestinal development and homeostasis and elucidate the pathogenesis of intestinal disorders, including unidentified diseases, several *in vitro* models have been developed. Human pluripotent stem cells (PSCs), including embryonic stem cells (ESCs) and induced pluripotent stem cells (iPSCs), have remarkable developmental plasticity and possess the potential for a wide variety of applications. Three-dimensional organs, termed *organoids* and produced *in vitro* by PSCs, contain not only epithelium but also mesenchymal tissue and partially recapitulate intestinal functions. Such intestinal organoids have begun to be applied in disease models and drug development and have contributed to a detailed analysis of molecular interactions and findings in the synergistic development of biomedicine for human digestive organs. In this review, we describe gastrointestinal organoid technology derived from PSCs and consider its potential applications.

## Introduction

To understand intestinal development, homeostasis and reproduction, and elucidate the pathogenesis of intestinal disorders, including infection, malabsorptive diarrhea, inflammatory bowel disease, digestive system neoplasia, and congenital disease, many studies have been conducted to generate tissues of the gastrointestinal tract, one of the complex organ systems in the human body. Researchers recently have made efforts to manufacture three-dimensional (3D) organs, called *organoids*, *in vitro* by imitating their genesis and have started to apply these to disease models and drug development. This paper outlines the characteristics of gastrointestinal tract, pluripotent stem cells (PSCs) and intestinal organoids derived from PSCs and demonstrates how intestinal organoids contribute to understanding of the processes of morphogenesis and diseases.

### Development of the gastrointestinal tract

For humans, the primitive gut tube, which has the capacity to differentiate into the mature gastrointestinal tract, appears as a straight tube during the fourth week of pregnancy and is divided into three segments (foregut, midgut and hindgut) along the anterior to posterior axis. Accompanied by the development of the fetus, repeated gut rotation and fixation (dynamic movements) and genetic signaling from endoderm and mesoderm tissues help to establish the esophagus, stomach, the small intestine (duodenum, ileum and jejunum), the large intestine (ascending colon, transverse colon, descending colon) and rectum ^(1)^. The morphogenesis of villi and crypts occurs, and the small intestine of a fetus subsequently becomes absorptive by taking in amniotic fluid by the 13th week of pregnancy ^(2)^. The length of the intestine increases along with the growth in body length, and a rapid rate of prenatal small intestinal growth ensures an adequate small intestine to satisfy postnatal nutritional requirements for the mature newborn ^(3)^.

### Histological structure and function of the intestines

Histologically, the gastrointestinal wall consists of three layers from the lumen outwards: mucosa, muscular layer, and serosa. The mucosa is made up of four layers: an epithelial layer of mucosa, lamina propria, lamina muscularis mucosae and submucosa. The muscular layer underlying the submucosa plays a role in peristaltic movements caused by smooth muscle (circular and longitudinal muscles) and enables foodstuffs to be propelled along the gastrointestinal tract to be assimilated as nutrients. In the lamina propria, lymphoid follicles constitute gut-associated lymphoid tissue (GALT) and aggregate to form Peyer’s patches (PPs) ^(4)^. PPs and isolated lymphoid follicles form a specialized immune system of the gut, harboring approximately 70% of peripheral immunocytes and comprising the largest lymphoid tissue in the body ^(5)^. In submucosa and muscular layers, two major neural plexuses are present (submucosa: submucosal plexus and muscular layer: myenteric plexus). The enteric nervous system (ENS) is found embedded along the full length of the intestinal tract to form meshes. The ENS orchestrates an independent neural network and regulates many functions, such as peristaltic movement, blood flow, secretion, absorption and more ^(6)^.

The epithelial surface of the small intestine forms circular folds, where mucosa protrudes into the lumen with submucosal tissue to increase the absorption efficiency of nutrients. Moreover, circular folds consist of numerous microvilli, hair-like structures that amplify the epithelial surface of the intestinal tract interior. This interior is covered with a single mucosal layer and an epithelial surface area of 30 m^2^ that was thought hitherto to measure 260–300 m^2^, almost equivalent to the area of a tennis court ^(7)^. Thus, the wide absorptive area of the small intestine enables efficient nutrient absorption. The lumen of the duodenum is characterized by long and well-developed villi, which become gradually shorter and flatter in the jejunum and ileum and are not found in colon. The small intestine is responsible for not only nutrient absorption but also metabolism; it also functions as an endocrine gland and in immunoregulation. Moreover, a physical barrier formed by intestinal epithelial cells separates the injurious luminal environment from the underlying intestinal layers ^(8)^. The mucosal epithelium of the small intestine is one of the tissues with the highest proliferation and regeneration potential in our body. In the mouse, the small intestinal epithelium entirely turns over every 5 days, maintaining dynamic homeostasis and structural integrity.

It is necessary to elucidate pathophysiology of diseases relating to intestinal disorders by demonstrating differentiation hierarchy in humans, and it is required to construct a species-specific model that mimics the process of gastrointestinal development and the biological tissue structure in an in vitro culture system. Recent advances in organoid technology are expected to deepen understanding of complex intestinal functions and tissue homeostasis and contribute to research on disease mechanisms.

## Generation of Intestinal Organoids in a Dish

### Dynamic regeneration of the intestines *in vitro*

The mucosal epithelium of the small intestine consists of villi and crypts, excluding regions adjacent to PPs. In a unit of a villus and a crypt, cells are brought into existence in the crypt component and become subdivided into various types of cells that subsequently migrate to the top of the villus, as on a belt conveyer. In mucosal epithelium, six major differentiated cell lineages exist that can play a variety of functions. Enterocytes that account for 90% of the epithelium perform functions that allow the absorption of nutrients from digested food and protect the integrity of the epithelium through structural properties and antigen uptake ^(9)^. Secretory lineages include goblet cells that produce mucus, endocrine cells that secrete hormones, Paneth cells that produce antimicrobial peptides and configure the stem cell niche, tuft cells that have a defensive effect against parasitic infection, and microfold (M) cells that take up intestinal microbial antigens and hand them over to GALT in the mucosal epithelium. The dynamic and continuous regeneration of mucosal epithelium was noted in the 19th century; moreover, a theory that crypt base columnar (CBC) cells between Paneth cells promoted differentiation of mucosal epithelial cells in crypt base compartments, called the unitarian hypothesis, was proposed ^(10), (11)^. By contrast, Potten and colleagues advocated that the stem cells retaining DNA labels for a long period located at position +4 relative to the crypt bottom, directly above the Paneth cells ^(12)^. After that, a great deal of effort has been devoted to studying and verifying “true” intestinal stem cells. Detailed studies analyzing molecular mechanisms in colorectal cancer have revealed that Wnt signaling and β-catenin/T-cell factor activity are crucial for regeneration and maintaining homeostasis of the mucosal epithelium. Such studies predicted that the expression of leucine-rich repeat-containing G-protein-coupled receptor 5 (Lgr5) was restricted by the Wnt target gene within crypts and was a cell marker expressed in CBC cells ^(13), (14)^. Lgr5-positive CBC cells were demonstrated to be the stem cell of the small intestine and colon and have the ability to self-replicate and the multipotency to generate all lineages ^(15)^. This discovery had a great impact on our understanding of the stem cell niche at the molecular level. This niche includes the Wnt agonist R-spondin ^(16)^, leucine-rich repeats and immunoglobulin-like domain 1 (Lrig1) as a negative feedback regulator of the epidermal growth factor (EGF) receptor family ^(17)^, notch signaling essential to the differentiation of enterocytes ^(18)^, and bone morphogenetic protein (BMP) signaling involved in crypt–villus formation ^(19)^. At the crypt bottom can be found Lgr5-positive CBC cells and adhering Paneth cells, differentiated by Wnt signaling, that secrete Wnt, EGF and notch essential for the regulation of dedifferentiation and differentiation. These signals are involved in the development of the small intestine’s mucosal epithelium. Furthermore, cell-lineage tracking experiments have revealed a cell differentiation hierarchy and that cellular plasticity exists because of the response of the stem cell niche. A dynamic cell differentiation hierarchy is of extreme importance for an appreciation of the complexity of intestinal disorders in humans. Therefore, it is necessary to construct *in vitro* models recapitulating the living body and developing processes of the digestive tract.

### PSCs and *de novo* organogenesis *in vitro*

In contrast to tissue stem cells that differentiate into a plurality of kinds of cell groups configuring a particular tissue, PSCs have the ability of unlimited proliferation from all three germ layers. Human PSCs, such as embryonic stem cells (ESCs) and induced pluripotent stem cells (iPSCs), have been demonstrated to proliferate indefinitely and differentiate into cells of any of the three germ layers ^(20), (21)^. Human ESCs and iPSCs have remarkable developmental plasticity and thus possess a great potential for human organogenesis, disease modeling and drug screening. They also have potential applications in regenerative medicine as a source of cell-based therapy ^(22)^. Work on PSCs focused on isolating particular cell types from differentiation at a two-dimensional (2D) level. However, in the human body, organ systems function through the integration of each system. Thus, the recapitulation of organogenesis is important for not only understanding fundamental mechanisms of human development but also translational applications in replacement therapy and in disease modeling. Tissue homeostasis is strictly organized under a tissue stem cell hierarchy. One of the well-characterized tissue stem cell models is intestinal stem cells. Active cycling of intestinal LGR5+ stem cells balances the maintenance of stem cells and the regeneration of mucosal epithelium cells to maintain epithelial homeostasis ^(23)^. Notable pioneering works by Sasai’s group have allowed the development of 3D culture methods using PSCs to recapitulate tissue organization in a dish ^(24), (25), (26)^. The reconstitution of a 3D tissue structure from stem cells has led to the use of the term “organoid.” This refers to 3D structures grown from stem cells that consist of organ-specific cell types that self-organize through cell sorting and spatially restricted lineage commitment ^(27)^.

### Generating human intestinal organoids

With regard to gastrointestinal organoids, these have two different cell sources: tissue stem cells (LGR5+ stem cells) and PSCs (Table 1). Sato’s fascinating work in 2009 indicated that isolated Lgr5+ stem cells from intestinal crypts generated 3D and self-organizing luminal structures and epithelium-only organoids after the addition of laminin-rich Matrigel and growth factors, which mimicked the intestinal niche *in vivo* ^(28)^. The initiation of investigations on intestinal organoids were specifically based on the lineage-specific embryological tracking experiments of mice models in Clevers’ laboratory ^(29)^. They succeeded in establishing a 3D structure (mucosal epithelial organoids) *in vitro* that consisted of multifarious types of small intestinal mucosal epithelium differentiated from self-organizing Lgr5-positive CBC cells to reproduce crypt–villus formation ^(16)^. Further studies of mucosal epithelial organoids have progressed and enabled the establishment of organoid culture conditions for epithelial stem cells from different species, including humans and mice, and different gastrointestinal sections, such as the stomach, the small intestine and the colon ^(30), (31), (32)^.

**Table 1.**
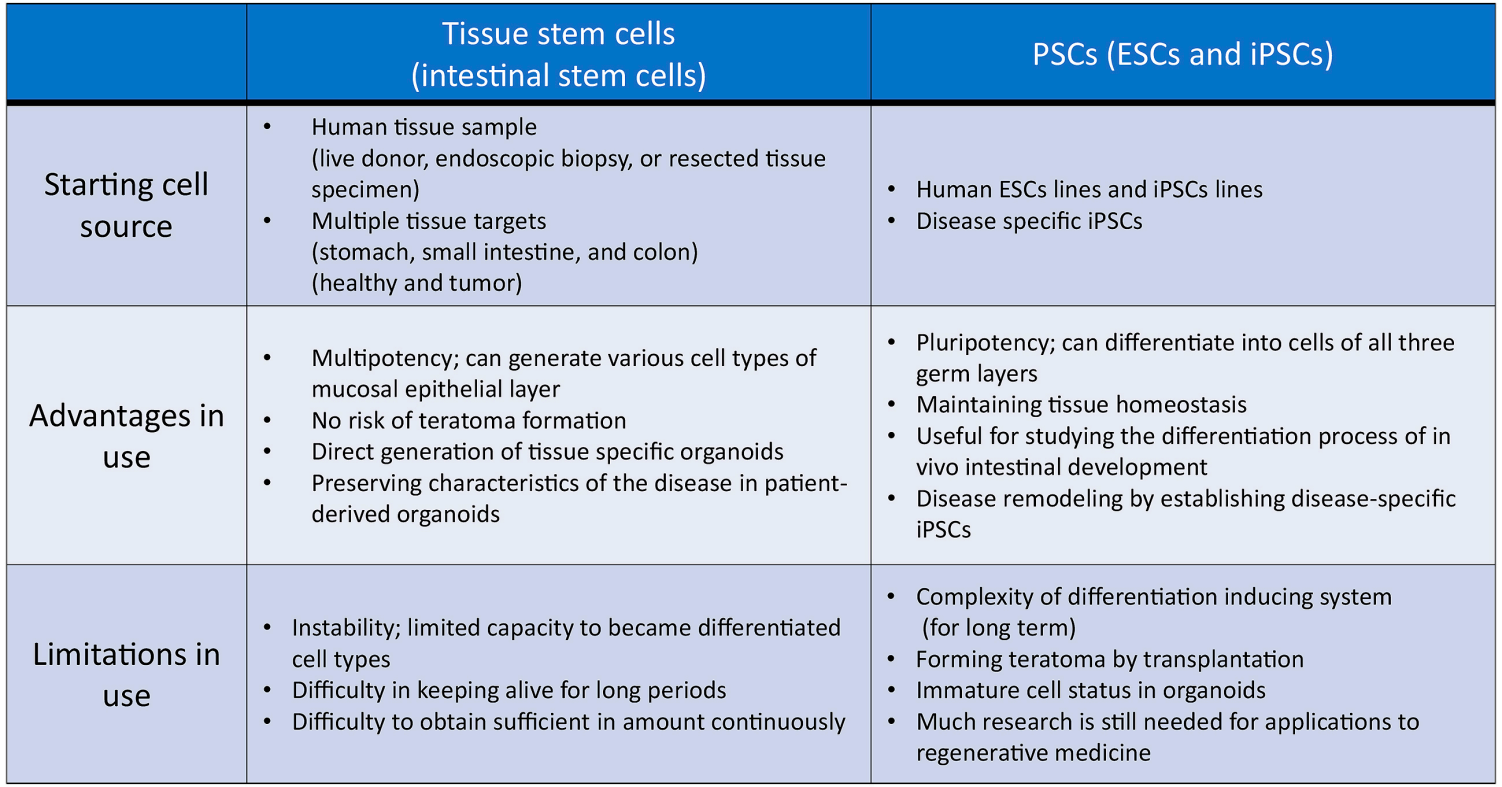
Usage of Gastrointestinal Organoids. PSCs; pluripotent stem cells, ESCs; embryonic stem cells, iPSCs; induced pluripotent stem cells

Subsequently, another method of directly differentiating human ESCs and iPSCs into human intestinal organoids (HIOs) was invented by Spence et al. ^(33)^. Intestinal organoids exhibit the following features: 1) They are comprised of a variety of cell types that constitute a tissue structure resembling an intestine *in vivo*, and 2) they indicate a formative process whereby various cells differentiate from stem cells to accumulate and become shaped into a 3D structure on the basis of the self-organization of dividing cells into appropriate sites containing a niche of stem cells and an extracellular matrix. In generating intestinal organoids derived from ESCs and iPSCs, two major differences exist with the mucosal epithelial organoids mentioned above: (i) Undifferentiated PSCs can recapitulate the human intestinal developmental process, and (ii) the differentiation of not only mucosal epithelial tissue but also submucosal tissue becomes possible in theory. However, it is not easy to recapitulate the gastrointestinal tract, including submucosal tissue, because the developmental process from the archenteron is quite complex. In addition, tridermic cells and tissues are interrelated and provide gastrointestinal functionality; therefore, methods mimicking the gradual differentiation of the early embryo were employed for inducing differentiation ^(33), (34)^.

Intestinal development begins with the formation of the embryonic endoderm germ layer that gives rise to the intestinal epithelium during gastrulation. This requires the *nodal* signaling pathway that controls the expression of crucial transcription factors ^(27), (35)^. Definitive endoderm presents as a 2D sheet of cells and subsequently forms a 3D primitive gut tube functionally subdivided along the anterior–posterior axis into a foregut, midgut, and hindgut from which endodermal organs arise. In these processes, smooth muscles and the vascular system are derived from the mesoderm, and the ENS derived from the neural crest penetrates along the gut tube. CDX2 is an obligate posterior marker that allows differentiation into midgut and hindgut, giving rise to the small and large intestines. Therefore, Spence et al. used activin to direct the differentiation of human PSCs into definitive endoderm to induce posterior endoderm patterning and mid- and hindgut specification highly expressing CDX2 through activation of both Wnt and fibroblast growth factor signaling, allowing cells to take up a caudal/hindgut fate and form small spheroids. In addition to exposure to WNT3A/FGF4 as hindgut-specific growth factors, collecting and embedding hindgut patterning spheroids into Matrigel droplets, and passaging and maintaining them in tissue-specific growth factors (Noggin, EGF, and R-spondin) enabled the spheroids to differentiate into HIOs ^(33), (36)^.

Generating HIOs from human PSCs requires the modification of an additional factor for each stage and 3D suspension and self-assembled culturing in a Matrigel droplet. HIOs contain a luminal component lined with many columnar epithelial cell types of the intestine, including enterocytes, goblet cells, Paneth cells and enteroendocrine cells, as well as supporting mesenchymal clusters, including fibroblasts, myofibroblasts and smooth muscle cells. The coincident differentiation of epithelial and mesenchymal tissues suggested that interaction between these tissues may be important in the developmental process of PSC-derived intestinal organoids. In the closed luminal structure of HIOs, mucosal epithelium exists that has polarity and exhibits introversion; enterocytes having apical microvilli and peptide absorption ability, that is, HIOs, have an epithelium structure and function that resembles the small intestine, although they are not mature ^(33)^. Since deep crypts and the expression of the intestinal stem cell marker, Lgr5, were not found in HIOs, they are regarded as persisting in a transcriptional and functional state similar to fetal human tissue ^(37), (38)^. As a feature of cell culture, HIOs are able to be cut into small pieces and passaged with specific growth factors *in vitro* for up to a year ^(33), (38)^. Moreover, the size of HIOs is only approximately 2 mm, which demands cogitating to use HIOs in various evaluations ^(39)^.

### Current limitations of HIOs

Several limitations exist regarding the use of HIOs as a human small intestine *in vitro*. First, HIOs do not contain the ENS. In the early developmental process, neural crest cells migrate into the mesenchyme along the digestive tube and form the ENS, which consists of more than 100 million enteric neurons and 400 million glial cells ^(40), (41)^. The neurons and glial cells are organized into the inner submucosal plexus and the outer myenteric plexus, regulating intestinal peristalsis movement with intestinal cells of Cajal that operate as pacemaker cells ^(42)^. Although HIOs contain mesenchymal cell types, including fibroblasts, smooth muscle and myofibroblasts, they do not have nerve plexuses and the interstitial cells of Cajal. The ENS comprises a functional intestinal neuronal–glial epithelial unit with intestinal epithelial cells, becoming a key regulator of the intestinal epithelial barrier function and of bowel movements ^(43)^. For *in vitro* studies on the role of gut microbiota using intestinal organoids, engineering of additional functional HIOs is required.

Another missing structure in HIOs is the immune system, which is one of the major functions of the intestinal tract. The trachea and the gastrointestinal tract are organs that contact the outside and in which mucosal lymphatic tissue develops. In the small intestine, PPs consist of lymphatic follicles, T cells, B cells, macrophages, and dendritic and M cells. M cells are a subset of intestinal epithelial cells, reside in the region covering PPs, and take up luminal antigens and present them to PPs for subsequent intestinal immune responses ^(4), (43)^. Although HIOs do not contain immune cells, including M cells, their applications as experimental models of mucosal epithelial barrier and intestinal infection have been reported. Thus far, the disruption of the intestinal mucosal epithelial barrier by *Clostridium difficile*, *Salmonella enterica* and *Escherichia coli* O157 is substantiated using HIOs as a bacterial infection model ^(44), (45), (46)^. In terms of viral infection models, although a method is lacking to construct efficient models that allow the infection of cultured cells or animal intestine with clinical strains of noroviruses, enteric adenoviruses and rotavirus, a study whereby clinical retrovirus isolates were replicated in HIOs was conducted by Finkbeiner et al. ^(47)^. The use of intestinal organoids as transformative new tools for human virus studies will facilitate an understanding of human viral pathogenesis and advance the development of vaccines, antivirals and therapeutics ^(48)^.

The interaction between the intestinal microflora, including commensal and symbiotic bacteria and various cell types, in the intestinal tract constitutes not only regional but general homeostasis crucial for human health ^(49)^. A failure of this intestinal ecosystem has been suggested in the etiology and morbidity of intestinal diseases such as inflammatory bowel disease and other systemic diseases ^(50)^. In these fields, intestinal organoids are expected to be used as a new biomodel of the host–microbe interface ^(51)^.

Furthermore, applications of model organisms to observations of congenital gut disorders *in vitro* have been attracting increasing attention. HIOs were derived from stem cells with a deleted endogenous *NEUROG3* candidate gene, responsible for congenital malabsorptive diarrhea and lost intestinal enteroendocrine cells. These demonstrated that NEUROG3 expression contributed to the differentiation of enteroendocrine cells. A decrease in the number of mature enteroendocrine cells is related to the development of this disease ^(33)^.

Taken together, HIOs are expected to be applied to investigations of human intestinal infection, which were not able to be undertaken using experimental animals, as well as in the development of medicines and in disease research. However, problems exist in generating fully functional intestinal tissue containing a mesenchymal tissue structure and the immune system ^(39)^. The *in vivo* transplantation of HIOs under the kidney capsule in mice enabled the maturation of the epithelium and mesenchyme of such HIOs compared to those generated *in vitro* ^(52)^. In addition, HIOs containing a functional ENS were generated by co-culturing with human PSC-derived neural crest cells, which differentiate into glial cells and neurons, that are used for investigations of the cellular and molecular basis of Hirschsprung's disease ^(53)^.

### Establishing another intestinal organoid “Mini-Gut” *in vitro*

**Figure 1.**
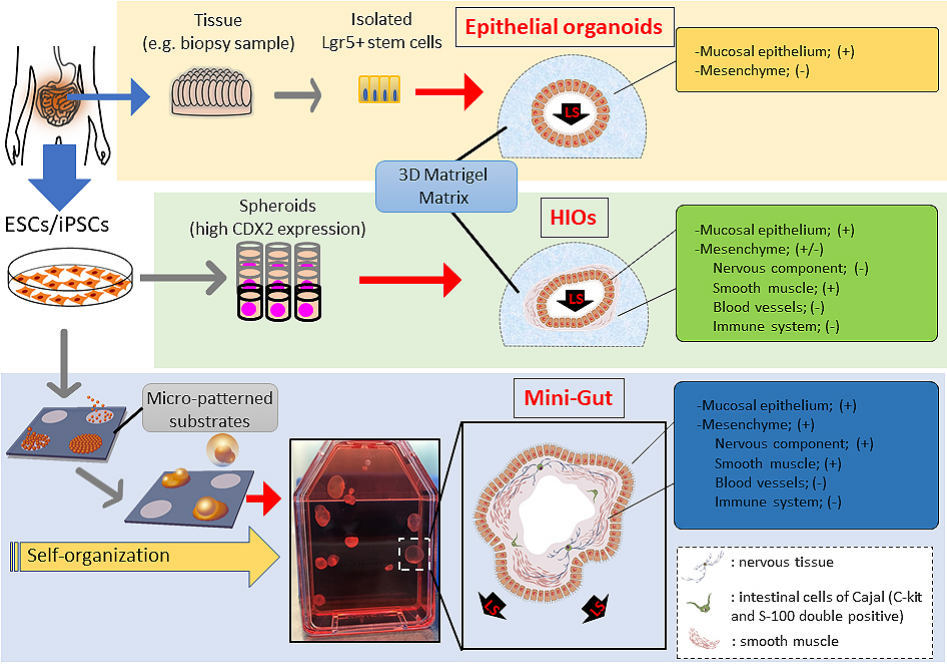
Intestinal organoids are generated from either isolated intestinal stem cells or human embryonic stem cells (ESCs)/induced pluripotent stem cells (iPSCs) *in vitro*. Lgr5+ cells isolated from collected tissue are embedded in a three-dimensional (3D)-Matrigel suspension culture and grow into organized epithelial organoids. Pluripotent stem cells (PSCs) differentiate into CDX2 expressing spheroids containing endoderm and mesoderm and subsequently grow in Matrigel (HIOs). Mini-Guts are self-organized from PSCs in a xenogeneic-free culture condition. The Mini-Guts contain intestinal epithelium and mesenchymal layers, with the epithelial layer as an outer layer. LGR5+, leucine-rich repeat-containing G-protein-coupled receptor 5 positive; LS, luminal side.

In recent years, our group generated a new type of intestinal organoid, which is known as the “Mini-Gut,” consists of all three germ layers, and displays several of the intestinal physiological functions of human ESCs or iPSCs ^(54)^. Mini-Gut differs from conventional intestinal organoids in several ways relating to its production and properties. Mini-Gut is directly differentiated from human PSCs under xenogeneic-free conditions, without animal-derived components such as Matrigel and may be preferred in clinical applications. Furthermore, the production of Mini-Gut mimics the functional interaction and self-assembly of the three primary germ layers from an initial stage of culture on micro-patterned substrates. This differs from HIO culture in which endoderm is first generated using the serial induction of growth factors; the differentiation of specific intestinal cell types and other germ layers is subsequently induced. After day 30, Mini-Gut is observed as self-organized cystic spheroids that begin to spontaneously detach from the plate. It has a cystic tissue structure, with the mucosal surface side facing outward and mesenchymal tissue surrounding the lumen on the inside (Figure 1). Although Mini-Gut cannot be decomposed mechanically or enzymes used for passaging, Mini-Gut can persist for approximately a year as a single unit. It is proposed that Mini-Gut maintains its own tissue homeostasis, whereas a more precise examination is required regarding the cell cycle and differentiation mechanism of LGR5-positive cells.

The extroversion of the mucosal epithelium of Mini-Gut is morphologically unique, unlike other intestinal organoids, facilitating its manipulation in experiments of chemical substances or drugs and the testing of intestinal infectious agents, including bacteria and viruses. Sizes of Mini-Gut are 10 mm or larger, which enables the visual viewing of metabolic and absorptive performance in experiments using fluorescent materials. Moreover, mesoderm-derived smooth muscle cells and ectoderm-derived intestinal nerves are present in Mini-Gut, displaying a contractile activity in response to treatment with histamine and atropine, similar to the peristalsis movements of mature human intestine. This organoid contains intestinal neurofilaments and is expected to be used in studies of Hirschsprung’s disease, in which gangliocytes do not exist because of a congenital defect, and related disorders that possibly come from the functional failure of gangliocytes. As with mucosal epithelial organoids and HIOs, Mini-Gut generated from human PSCs has the properties of intestinal organoids. It is anticipated that Mini-Gut will have several medical uses, including in research on the development of the human intestine, homeostasis, local and systemic diseases, and pharmaceutical agents (Table 2).

**Table 2.**
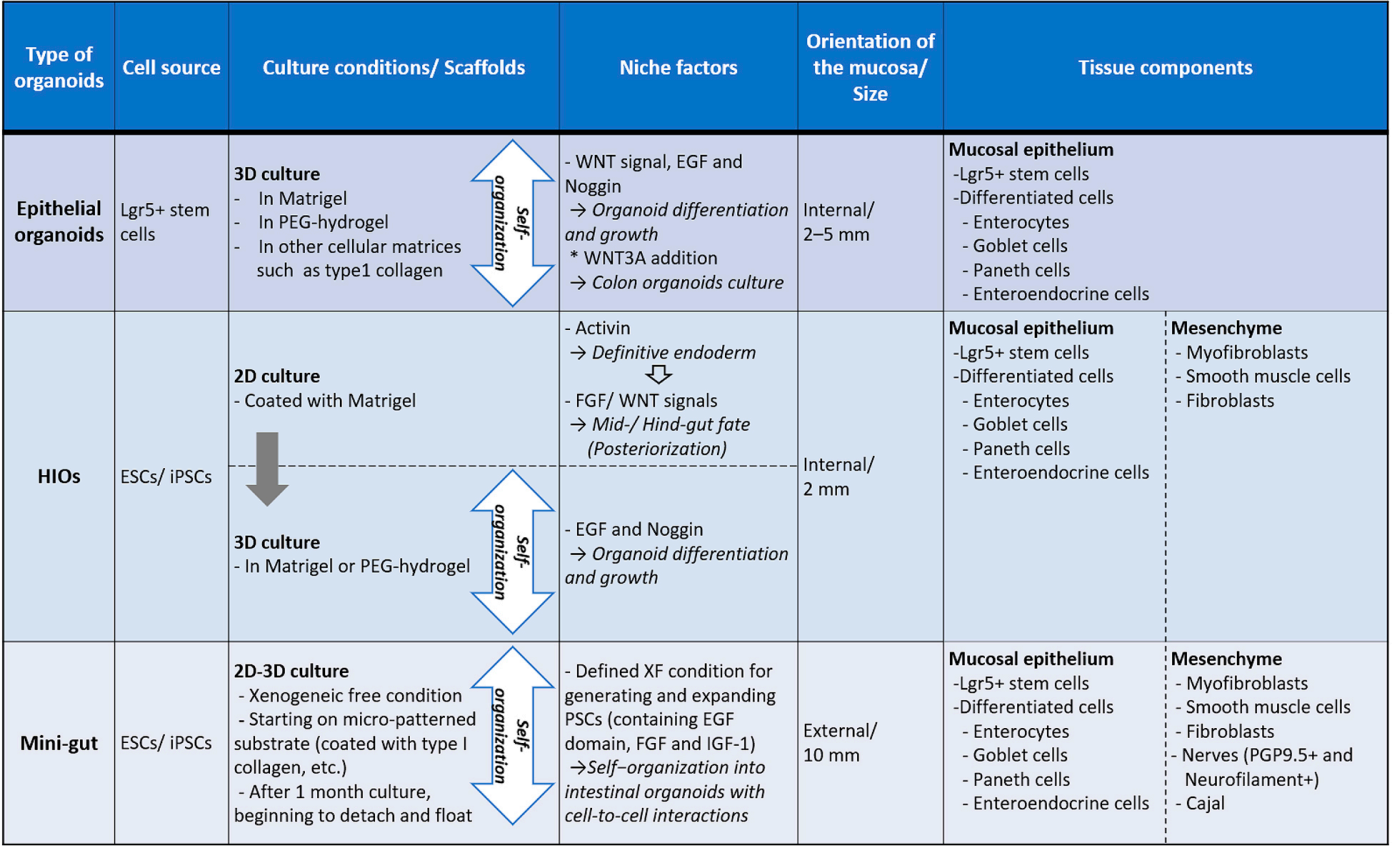
Characteristics of Intestinal Organoids. HIOs, human intestinal organoids; ESCs, embryonic stem cells; iPSCs, induced pluripotent stem cells; PSCs, pluripotent stem cells; PEG, polyethylene glycol

## Future Directions

### Challenges of intestinal organoids for the next applications

**Figure 2.**
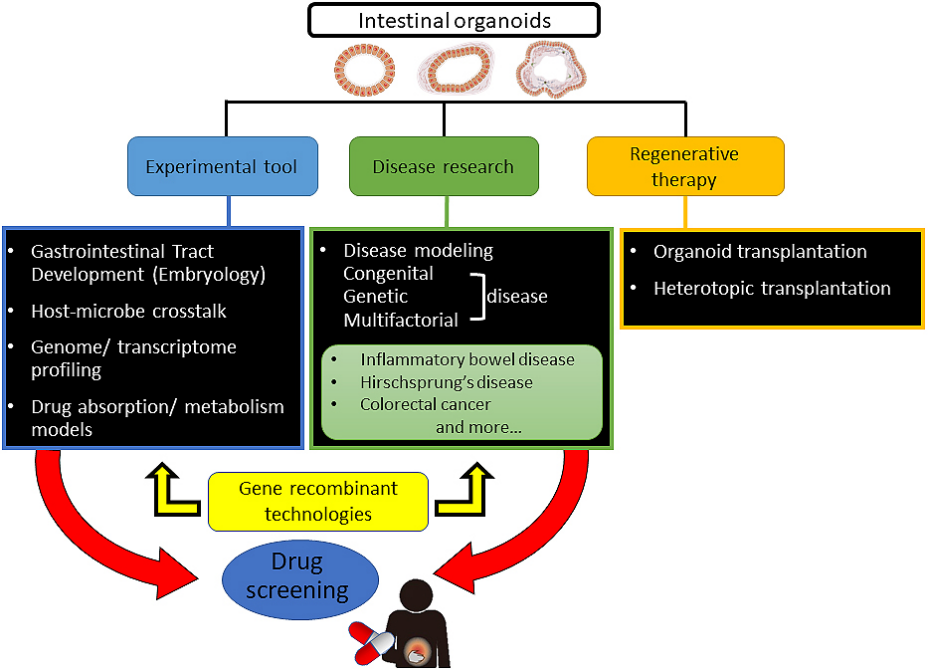
Applications of intestinal organoids. Intestinal organoids provide new opportunities for studying development of the gastrointestinal tract, host–microbe crosstalk and human diseases including inflammatory bowel disease, Hirschsprung’s disease, and colorectal cancer. With genome and transcriptome analysis, organoids reflect individual drug absorption and metabolism and can be used for drug screening, leading to the development of personalized treatment strategies. Gene recombinant technologies can also be exploited for studying specific genetic alterations and signaling pathways and making disease models. The study of organoid transplantation and maturation is also in progress, to use organoids in regenerative medicine.

Presently, intestinal organoids generated from epithelial stem cells or PSCs are used in basic and disease research and in *in vitro* drug development as biomodels that recapitulate human intestines (Figure 2). Recently, new markers and cell types have been discovered in epithelial cells profiled from the mouse’s small intestine and organoids using a single-cell survey ^(55)^. For the purpose of application to human disease research, although organoid technologies will become extremely valuable, it is essential to understand the characteristics of both intestinal stem cells and PSCs, including their advantages and disadvantages, and to utilize proper evaluating methods (Table 1). Moreover, studies on the role of the microbiota and macrophages in intestinal physiological functions, homeostasis, immunity and systemic diseases are continuing ^(56), (57), (58), (59)^; if highly functional intestinal organoids are used, these will become an appropriate biotool. Model systems including culturing devices and techniques have also been developed to reproduce complex in vivo human intestinal tracts and provide a platform for host–microbe crosstalk research and drug discovery applications ^(60)^. For instance, to improve accessibility of microbial adhesion and drug addition to the luminal side and operability of reactivity evaluation on the basement membrane side, methods to reconvert 3D structure prepared in Matrigel into 2D (single-layer) structure using TransWell membrane had been established ^(61)^. The air–liquid interface methods have been reported to allow long-term culture of various differentiated cell types derived from collected murine intestinal stem cells ^(62)^. To expand the use of organoids that form 3D tissue structures and have tissue-specific functions in research and development, new imaging technologies and evaluation techniques are required. Using hitherto 2D-imaging systems, detailed information obtained from organoids cannot be sufficiently utilized. Because organoids exhibit continuous tissue homeostasis, a 4D rather than a 3D-imaging system will help in monitoring developmental and tissue structure changes, including sequential changes and increase the usefulness of intestinal organoids, making these unprecedented biomedical tools carrying disease-associated genetic information. Integrating visualized and disease models using genome editing technologies, intestinal organoids could become *in vitro* biomodels, surpassing existing models.

Intestinal organoids and PSC-derived organoids are also expected to be useful *in vitro* models for diseases such as enteric infections and gastrointestinal cancers. The organoids mimic the physiological condition between enteric pathogens and the host intestine and allow the analysis of the intestinal barrier function ^(63), (64)^. The intestinal organoid system could apply for the intestinal mucosal vaccine development. In gastrointestinal cancer research, the organoid culture technique imitates and reflects an *in vivo* tumor microenvironment. Particularly, the mucosal epithelial organoids enable single primary patient-derived organoids as preclinical cancer models. These cancer organoids could potentially propose a physiologically relevant reaction, compared with existing simple cancer cell lines, and are expected to be applied in individualized medical care and efficient drug development ^(65), (66)^.

### Expectations for regeneration therapy

Many types of diseases originate in the intestine and colon, including congenital, genetic and multifactorial diseases. Many diseases, such as inflammatory bowel disease and Hirschsprung’s disease, lack effective or fundamental medical treatments, resulting in either a significant reduction in the quality of life or threaten the life of patients. The intestine affects all parts of an individual organism, including nutrient digestion and absorption, metabolism, immunity and commensal microbiota. Therefore, investigation of the use of intestinal organoids as cell sources in regenerative therapy for a lack of functional intestine is required. Although the total number of performed intestine transplants has decreased over the past decade because of improvements in medical and surgical treatments, intestine transplants will still play an important role in the treatment of intestinal failure, notwithstanding the difficulty of immune activity regulation of the intestine ^(67)^. As a method that replaces allotransplantation, introducing mucosal epithelial organoids generated from autologous tissue stem cells or human PSCs derived from epithelial/mesenchymal organoids, including HIOs, to patients is anticipated, and research and development on it is being undertaken ^(68), (69)^. The generation of HIOs using the polyethylene glycol (PEG) hydrogel resolved a problem of using Matrigel extracted from mouse sarcoma in regenerative medicine ^(61)^. As a result of advances of the gastrointestinal tissue engineering, which promotes the maturation of intestinal organoids ^(53)^ and transplantation techniques of heterotopically engrafting small intestinal epithelial organoids into the colon ^(70)^, it may be possible to provide medical care that complements the lost intestinal function of the short bowel syndrome caused by extensive resection of the intestine due to necrotizing enterocolitis or inflammatory bowel disease.

## Conclusion

Research on PSCs, including human ESCs and iPSCs, has benefited developmental biological studies. Recently, the range of applications for PSCs has widened, including in disease modeling, pharmacological testing and regeneration, and cell transplantation therapy. Moreover, a combination of understanding the embryonic development of the digestive tract and the mechanisms that maintain homeostasis of intestinal mucosal epithelium have led to the innovative establishment of human gastrointestinal organoids generated from undifferentiated human PSCs. Investigations utilizing gastrointestinal organoids contribute to a detailed analysis of molecular interactions and result in synergistic development in these fields. Although intestinal organoids containing not only epithelium but also mesenchymal tissue that partially recapitulates the complexity of the intestine, more functional intestinal organoids generated from human PSCs are expected to promote further drug developmental studies and clinical regenerative therapies specifically targeted to patients.

## Article Information

### Conflicts of Interest

None

### Sources of Funding

This work was partially supported by a National Center for Child Health and Development Seiiku Medical Study Grant (grant number 30-4) and the Danone Institute of Japan Foundation general researchers grant (grant number DIJF H30-036) to HA.

### Acknowledgement

The authors thank their funding sources: the Danone Institute of Japan Foundation and the National Center for Child Health and Development. We also thank Tomoyuki Kawasaki for figure illustration and laboratory colleagues for their helpful discussions.

### Author Contributions

Satoru Tsuruta and Hidenori Akutsu made substantial contributions to the conception and design of the work and drafted and revised it critically for important intellectual content. Hajime Uchida made contributions to the conception of the work.

### Approval by Institutional Review Board (IRB)

This manuscript is a review article and did not need approval by an IRB.
